# Adapting Clinical Tooth Wear Assessment Methods for Biological Anthropology Contexts

**DOI:** 10.1002/ajpa.70080

**Published:** 2025-06-24

**Authors:** Ian Towle, Luca Fiorenza

**Affiliations:** ^1^ Biomedicine Discovery Institute, Department of Anatomy and Developmental Biology Monash University Melbourne Australia

**Keywords:** craniofacial growth, dental attrition, dental tissue loss, digital anthropology, *WearCompare*

## Abstract

**Objectives:**

Tooth wear is increasingly recognized as an adaptive process that can help optimize mastication and maintain oral health. In this study, we apply clinical wear‐assessment methods to quantify occlusal tissue loss in first molars of seven Australian Aboriginal individuals from Yuendumu (1950s–1970s), whose diet combined traditional hunter‐gatherer foods with processed Western items.

**Materials and Methods:**

High‐resolution surface scans of dental casts were analyzed using *WearCompare* to assess wear patterns during dental development and evaluate the applicability of these tools in a biological anthropology context.

**Results:**

Clinical methods designed for assessing pathological wear can effectively capture normal physiological wear in populations with medium‐high tissue loss rates. Average annual tissue loss was 4 mm^3^ (0.05 mm^3^/mm^2^), with the highest wear regions losing an average of 215 μm in thickness per year. Substantial temporal variation in the magnitude and distribution of wear, and variation among individuals, was observed, reflecting changes in occlusion, masticatory forces, craniofacial growth, and cultural/dietary behaviors all in association with dental eruption sequences.

**Discussion:**

These findings highlight the utility of digital quantification of dental tissue loss for anthropological research. In particular, these methods have significant potential for assessing tooth wear in contemporary human and non‐human primate samples, and for refining macroscopic wear scoring systems in paleontological and archaeological contexts by using modern analogues for calibration and refinement. These methods can also complement other wear analysis techniques (e.g., microwear analysis, occlusal fingerprint analysis), as well as studies on tooth morphology and structure, offering broader applications in evolutionary inferences and dietary reconstructions.

## 1 Introduction

Tooth wear, the loss of dental tissue caused by attrition, abrasion, and erosion, has been extensively documented across diverse human populations (e.g., Molnar 1971; Eshed et al. 2006; Clement et al. 2012; Masotti et al. 2017; Witecy et al. 2021). In archaeological contexts, tooth wear analysis provides critical insights into dietary habits, behaviors, and environmental interactions (Esclassan et al. 2009; Fiorenza et al. 2018; Towle et al. 2023; Crété et al. 2024). Comparative studies between groups, such as hunter‐gatherers and agricultural populations, have found significant differences in wear patterns. Hunter‐gatherers typically exhibit more pronounced wear due to the consumption of coarse, unprocessed foods, whereas agriculturalists, consuming softer and processed diets, often show reduced wear. However, agricultural diets can often still lead to notable tissue loss, influenced by food processing techniques, accidental ingestion of grit, and the influence of caries or erosion (Smith 1984; Molleson and Jones 1991; Larsen 1995). In addition to providing dietary insights, tooth wear has been used for estimating the age of individuals in archaeological and paleontological studies (Nowell 1978; Helm and Prydsö 1979; Scott 1979; Mays et al. 2022). In both types of studies, wear is typically assessed using scoring systems that evaluate specific cusps, individual teeth, or entire dentitions.

Tooth wear is a natural and often adaptive process rather than inherently pathological (Kaifu et al. 2003; Benazzi et al. 2013; Towle 2019). Tooth wear can adjust occlusal surfaces to maintain efficient mastication, prevent impaction, and potentially reduce the risk of malocclusion and caries (Ungar and M'Kirera 2003; Kaidonis 2008). Although wear severity differs across human populations, the progression of wear across occlusal surfaces of molars follows consistent patterns, including the pattern and order of cusp rounding/flattening and the development of dentine exposure across occlusal surfaces, such as “functional” molar cusps (lingual cusps of maxillary molars; buccal cusps of mandibular molars) vs. non‐functional cusps (Lovejoy 1985; d'Incau et al. 2012; Towle et al. 2021; Mays et al. 2022). However, existing wear scoring methods capture only these broad trends and lack the resolution needed to measure tissue loss across occlusal surfaces through time or identify periods of accelerated or slowed wear (i.e., phasic tooth wear progression). These complexities are heightened during dental development, as changes in occlusal forces and contact sizes, tooth eruption, and craniofacial growth will substantially influence wear progression (Molnar, McKee, and Molnar 1983; Molnar, McKee, Molnar, and Przybeck 1983; Brown et al. 2011; Loomans et al. 2017). Gaining a further level of detail could therefore be an additional tool for dietary reconstructions in both contemporary and past populations.

This study adapts protocols and software originally developed for the clinical assessment of pathological wear (O'Toole et al. 2019a, 2020) to explore patterns of dental tissue loss during dental maturation in a population exhibiting medium‐high physiological wear. Using digitized dental stone casts from individuals in the Yuendumu Australian Aboriginal population, who consumed a mixed diet of traditional hunter‐gatherer and Western foods (Brown et al. 2011), we quantify and visualize occlusal wear progression in maxillary and mandibular first molars over time. Our primary objective is to evaluate the applicability of these methods in settings characterized by extensive physiological wear, in contrast to clinical samples in which these techniques were developed. By tracking wear from the onset of first molar occlusion through to third molar eruption, we offer a longitudinal perspective of tissue loss during dental development and assess the subtilty of wear patterns these techniques capture. For this we compare our longitudinal data with established single‐time‐point studies (e.g., Smith 1984; Kaifu et al. 2003; Kullmer et al. 2009; Towle et al. 2021; Fiorenza et al. 2022). Finally, we consider the broader implications of these, and similar, dental tissue loss approaches for applications in biological anthropology.

## 2 Materials and Methods

The Yuendumu dental cast collection, housed at the University of Adelaide, was utilized for this study (Brown et al. 2011). Originally compiled between 1951 and 1976 as part of a longitudinal study on Australian Aboriginal orofacial development by Barrett, Brown, and colleagues (Brown and Barrett 1973), this unique dataset provides a rare opportunity to analyze tooth wear progression over time (Molnar, McKee, and Molnar 1983; Mayhall and Kageyama 1997; Lee et al. 2021). The population represented in this collection experienced higher physiological (i.e., non‐pathological) rates of tooth wear than most contemporary populations due to their transitional dietary practices, shifting from a predominantly nomadic, hunter‐gatherer lifestyle to incorporating more Western (i.e., softer and processed) foods (Campbell and Barrett 1953; Middleton and Francis 1976; Molnar, McKee, and Molnar 1983; Corruccini et al. 1990; Brown et al. 2011). The Yuendumu collection is one of the most extensively studied dental datasets worldwide, with over 250 scientific publications (Brown et al. 2011). This makes it an ideal sample to test the applicability of methods developed for studying pathological tissue loss in a clinical setting, in populations with significant, yet non‐pathological, wear (i.e., physiological tooth wear).

Dental casts were scanned using a high‐resolution structured‐light scanner (Breuckmann smartSCAN3D C‐5) to create detailed 3D models (Fiorenza et al. 2018; Oxilia et al. 2018). The scanner achieved a resolution of 45 μm, ensuring precise capture of surface details. Multiple views of each cast were manually aligned using homologous points, following a best‐fit alignment protocol (Lee et al. 2021). Artifacts on the surface of the casts (e.g., saliva/food during molding or small air bubbles during the casting process) were rare and removed using Meshmixer (Autodesk Inc., San Rafael, CA, USA), utilizing tool sculpt “Brushes”.

Seven individuals were included in this study: five with only two sets of dental casts (i.e., one set of tissue loss data generated), and two individuals with casts molded every 3 years from ages 8 to 17 (i.e., three sets of tissue loss data generated). All samples included first molars for both upper and lower dental arches, with the left tooth used in all cases. Wear progression was analyzed using a purpose‐built freeware, *WearCompare* (LeedsDigitalDentistry, UK; https://leedsdigitaldentistry.com/wearcompare/), created for use in clinical dentistry as a way to assess pathological tooth wear in patients through time (Kumar et al. 2019; O'Toole et al. 2019a; O'Toole et al. 2020). Three‐dimensional meshes (STL format) from each time point were superimposed using the iterative closest point algorithm, followed by manual fine‐tuning using buccal and lingual surfaces (via a selection paint tool; O'Toole et al. 2019a, 2019b; O'Toole et al. 2020; Towle, Krueger, et al. 2024). These surfaces were used for alignment due to typically undergoing the least change over time, ensuring maximum possible accuracy. Following O'Toole et al. (2019a, 2020), at least 75% of points on these buccal and lingual surfaces (using the paint tool) had to be within 25 μm of each other for the sample to be included in the study.

**FIGURE 1:**
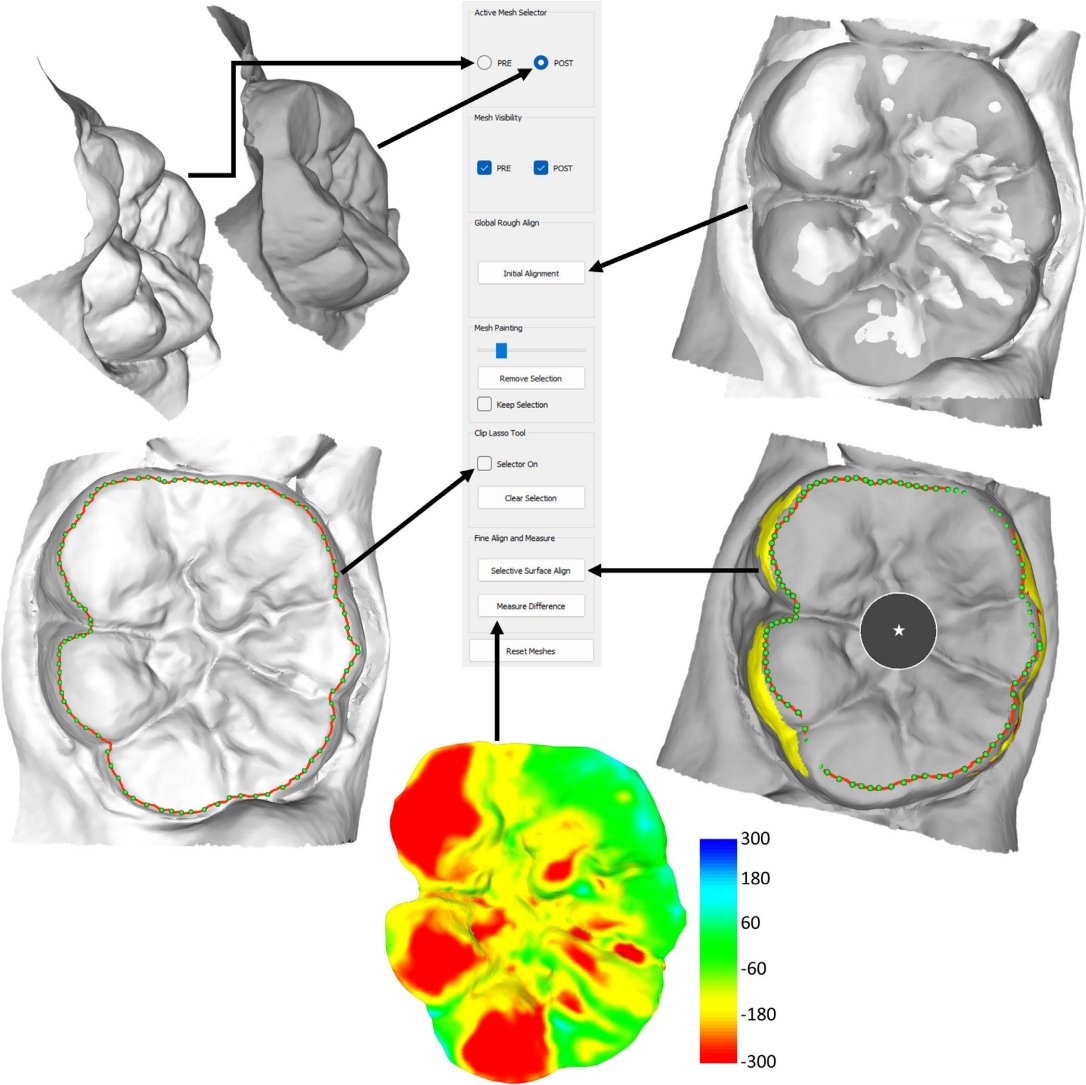
Overview of the *WearCompare* workflow. (1) Load both 3D models (STL files); (2) perform an initial global alignment of the two scans; (3) landmark the occlusal surface; (4) refine alignment using specific surfaces, here, the buccal and lingual surfaces; (5) define the direction for tissue loss calculation (in this case, perpendicular to the occlusal surface, indicated by the white star); and (6) generate tissue loss data and corresponding heatmaps. In this example, the heatmap ranges from +300 to −300 μm (default setting), though higher ranges may be more appropriate for individuals with greater wear. Small blue regions therefore represent minor artifacts or slight misalignments; however, in all cases analyzed here, these were minimal (i.e., limited surface area and < 100 μm depth). All images shown are of the mandibular left first molar from individual 634.

Using a landmark tool, the occlusal surface outline was traced and the direction of tissue loss specified (perpendicular to the occlusal plane). For each set of cast comparisons, three variables were calculated: total volume loss (mm^3^), volume loss per unit area (mm^3^/mm^2^), and maximum surface deviation (in microns). Figure 1 summarizes the entire procedure using the *WearCompare* software from initial global mesh alignment and refinement using the buccal and lingual surfaces, through using occlusal landmarking, to the subsequent calculation of wear metrics and generation of heatmaps. Average annual values were also calculated by dividing the tissue loss values by the number of years between moldings, and occlusal surface tissue loss heatmaps were compared to identify patterns of wear across different stages of dental development.

The use of the Yuendumu casts (from which the 3D models were generated) was approved by the Human Research Ethics Committee at the University of Adelaide (H‐27‐1990). The original ethical approval for the molding process required compliance with the National Health and Medical Research Council guidelines (Australia), which stipulated that the research be scientifically valuable, benefit the participants, respect the rights of the Warlpiri people, and not disrupt the routine of the settlement (Brown et al. 2011). Participants were informed about the study's aims and procedures, which involved no invasive techniques. Dental impression procedures involved commonly used dentistry methods at the time, with minimal risk of harm or discomfort (Brown et al. 2011).

## 3 Results

**FIGURE 2:**
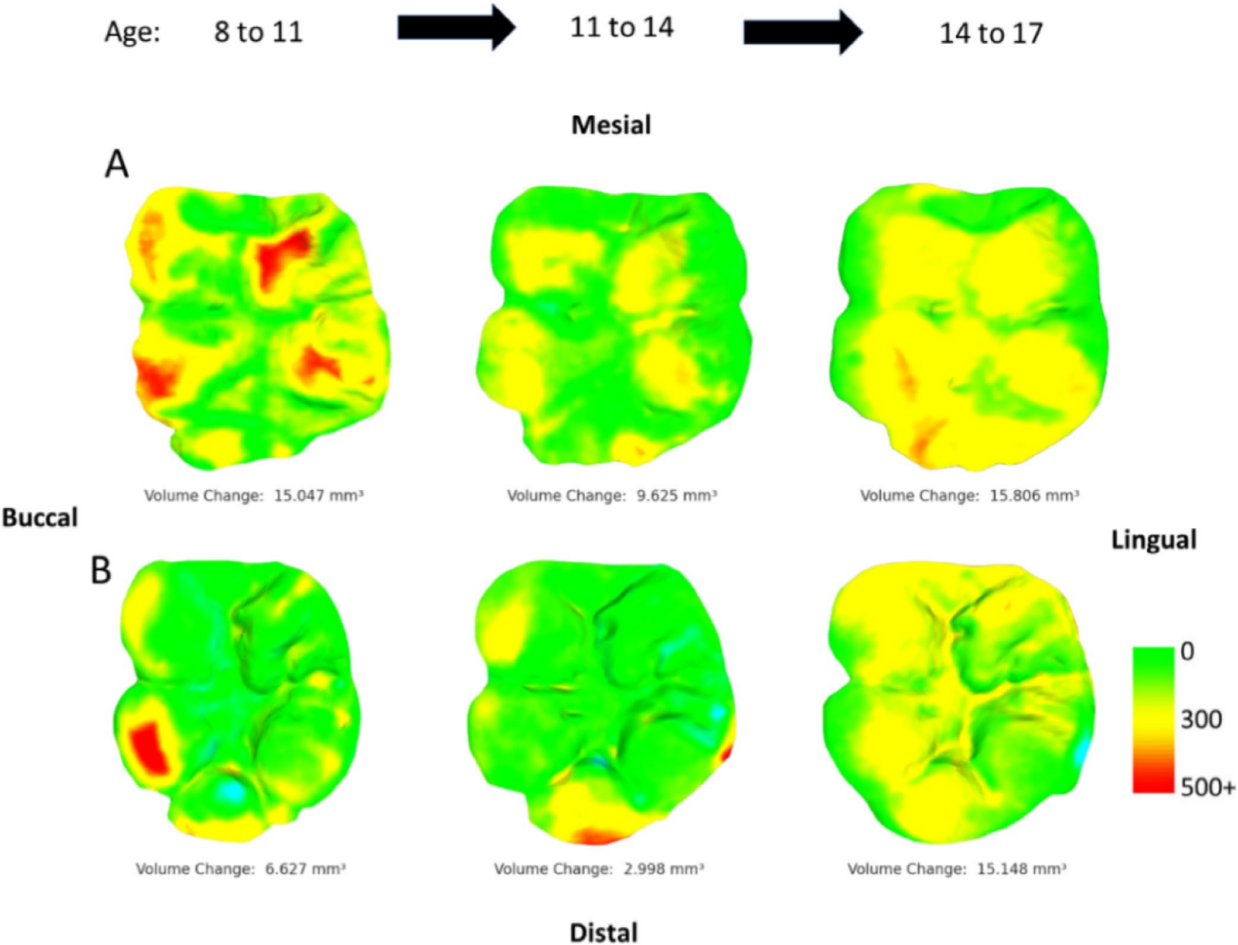
Visualization of tissue loss progression through time for mandibular first molars of individuals 549 (A) and 183 (B). Each set of images is ordered by time points from left to right. Scale in microns. Blue colouration indicates an erroneous “increase” in tissue (e.g., caused by saliva/debris during molding or slight alignment issues of the two casts).

**FIGURE 3:**
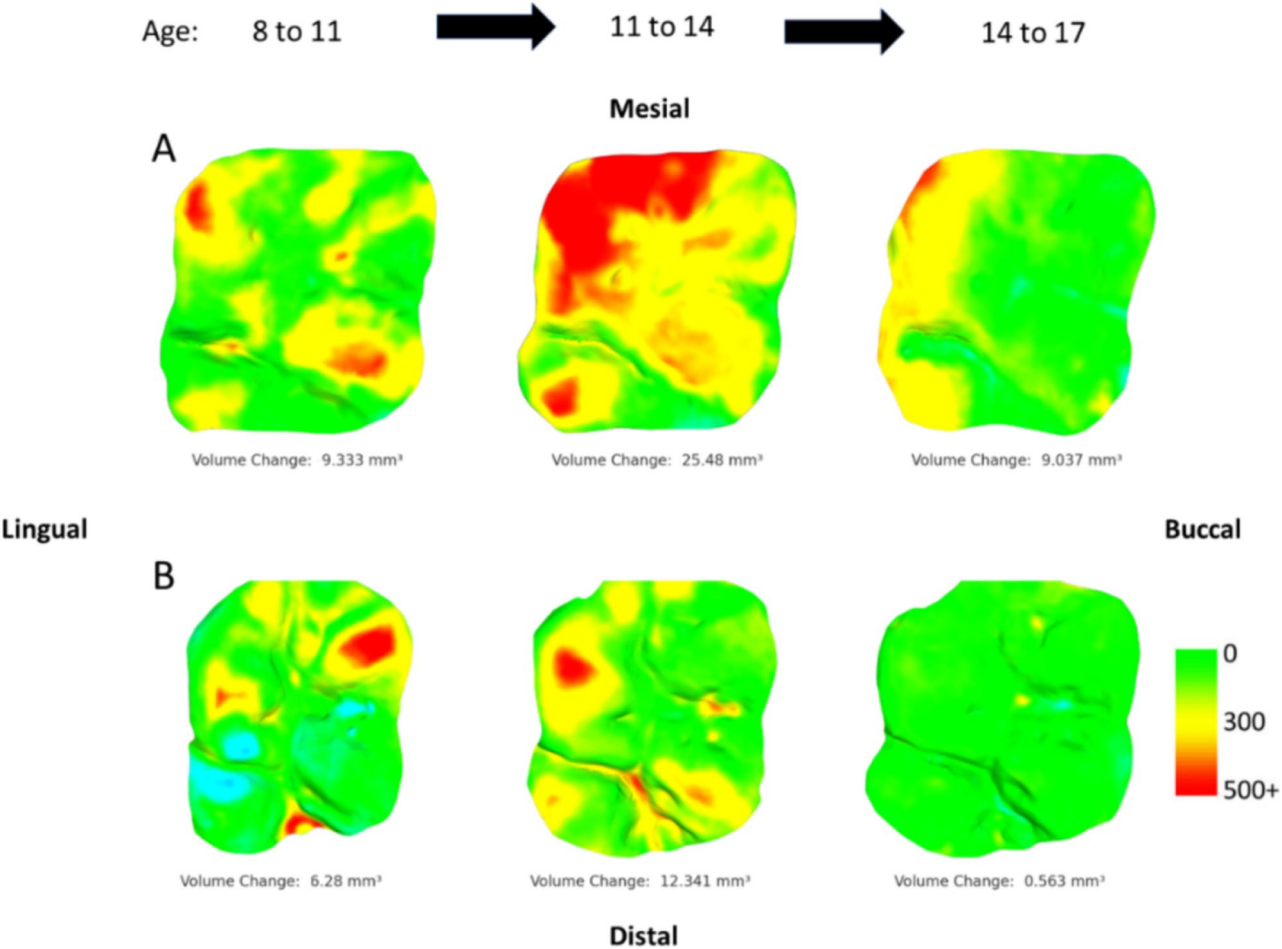
Visualization of tissue loss progression through time for maxillary first molars of individuals 549 (A) and 183 (B). Each set of images is ordered by time points from left to right. Scale in microns. Blue colouration indicates an erroneous “increase” in tissue (e.g., caused by saliva/debris during molding or slight alignment issues of the two casts).

**FIGURE 4:**
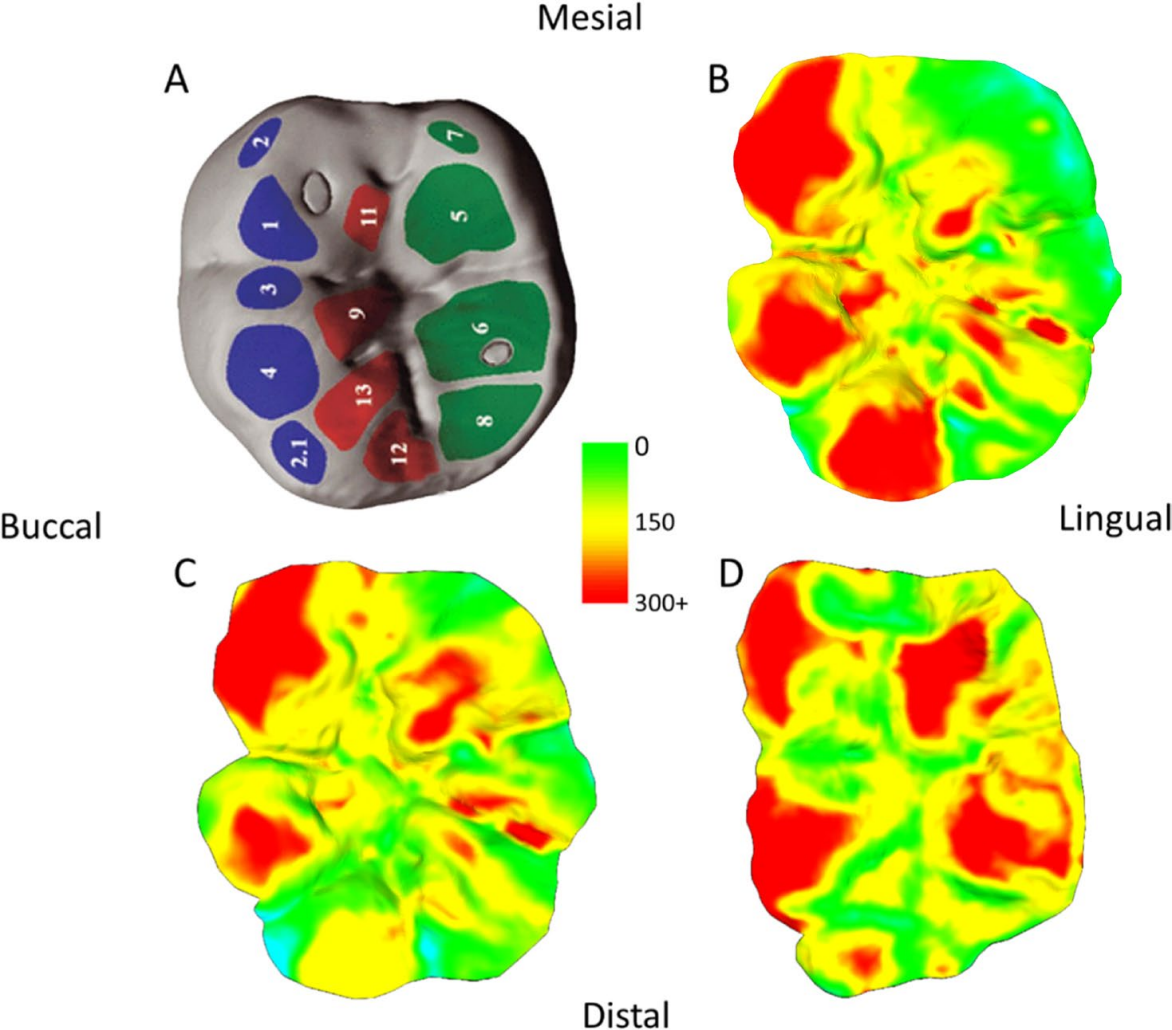
Visualization of tissue loss progression through time for mandibular first molars in comparison with areas often used for Occlusal Fingerprint Analysis (A; occlusal surface of a lower first molar with phase I (blue and green) and II facets (red) highlighted; source: Benazzi et al. 2011), for individuals 243 (B), 634 (C), and 549 (D). Each (B–D) represents occlusal tissue loss from approximate time of eruption of the first molar to 11 years old. Scale in microns. Small areas of blue colouration indicate an erroneous “increase” in tissue (e.g., caused by saliva/debris during molding or slight alignment issues between the two 3D meshes).

**FIGURE 5:**
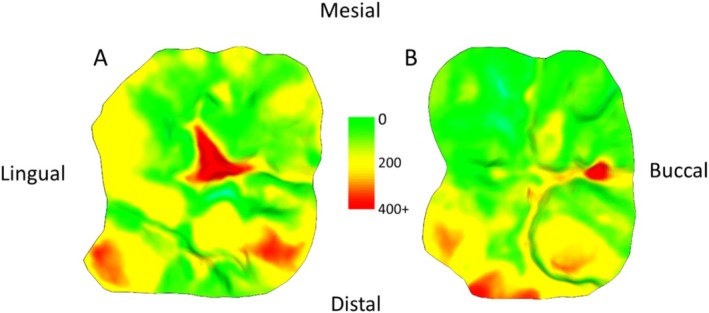
Individual 640, upper first molar (A) and lower first molar (B) showing tissue loss from the age of 8 to 9. Scale in microns. Note the potential for pathological tissue loss, since it is occlusal fissures and pits that show the highest degree of tissue loss, potentially relating to dental caries or acidic erosion. Small areas of blue colouration indicate an erroneous “increase” in tissue (e.g., caused by saliva/debris during molding or slight alignment issues between the two 3D meshes).

All 3D meshes were successfully aligned with their counterparts, indicating that the casts were suitable for this type of analysis, even with up to 3 years between moldings, as per alignment criteria established in clinical samples (i.e., > 75% of buccal/lingual data points were within 25 μm of each other; O'Toole et al. 2019b; O'Toole et al. 2020). There were no indications that these methods were unsuitable for medium‐high physiological tissue loss contexts, with minimal instances of erroneous tissue gain (e.g., due to misalignment or artifacts; see Figures 1, 2, 3, 4, 5). Combined with the high resolution of the casts and resulting 3D models, this allowed for the accurate visualization of even small‐scale changes in occlusal tissue loss between time points via occlusal heatmaps (Figures 2, 3, 4, 5). Tooth wear in all seven individuals was consistently high compared to clinical dentistry studies, with both upper and lower first molars showing substantial volume loss. The average annual tissue loss was 4 mm^3^ (0.05 mm^3^/mm^2^), and the highest‐wear regions in each tooth lost an average of 215 μm of tissue thickness annually. Females exhibited slightly more wear than males, and lower molars showed greater wear than upper molars (Tables 1 and 2).

**TABLE 1:** Dental tissue loss for upper first molars for each time point and individual studied.

Sample and age	Total volume loss (mm^3^)	Volume loss mm^3^/mm^2^	Maximum surface deviation (microns)
Individual 183 (male)			
8–11	6.28	0.06	588.10
11–14	12.34	0.11	497.60
14–17	0.56	0.01	211.50
Individual 549 (female)			
8–11	9.33	0.12	486.00
11–14	25.48	0.30	749.80
14–17	9.04	0.11	456.00
Individual 243 (male)			
8–11	3.68	0.04	418.30
Individual 251 (male)			
15–16	1.74	0.02	451.70
Individual 288 (male)			
8–11	3.48	0.04	361.00
Individual 634 (female)			
7–11	6.17	0.07	412.10
Individual 640 (male)			
8–9	10.96	0.13	786.60

**TABLE 2:** Dental tissue loss for lower first molars for each time point and individual studied.

Sample and age	Total volume loss (mm^3^)	Volume loss mm^3^/mm^2^	Maximum surface deviation (microns)
Individual 183 (male)			
8–11	6.63	0.07	605.40
11–14	3.00	0.03	570.80
14–17	15.15	0.14	411.20
Individual 549 (female)			
8–11	15.05	0.19	501.70
11–14	9.63	0.11	360.90
14–17	15.81	0.18	472.40
Individual 243 (male)			
8–11	9.96	0.11	529.80
Individual 251 (male)			
15–16	11.21	0.14	268.50
Individual 288 (male)			
8–11	1.56	0.02	238.60
Individual 634 (female)			
7–11	16.18	0.17	486.20
Individual 640 (male)			
8–9	9.88	0.12	534.30

There were notable differences among individuals and for the same individuals for different ages of dental development. For instance, the two individuals with the most extensive data (549 and 183) showed substantial, yet variable, tissue loss over time (Figures 2 and 3). Individual 549 exhibited substantially more tissue loss than individual 183, particularly in the upper molars. However, there are some consistent patterns between them, in particular, for both individuals the maximum tissue loss occurs in the 11–14 age range for the maxillary first molar, whereas the exact opposite pattern is found in the mandibular molars with this age point being the lowest tissue loss in both individuals.

For individuals with similar time points covered (i.e., comparable ages of molding), the wear across the occlusal surface follows a consistent pattern. These patterns align with known wear trajectories, such as “functional” cusps (buccal cusps of the lower molars and lingual cusps of the upper molars) exhibiting greater wear, and in many cases, they also seem to correspond well with established occlusal wear facets documented in the literature (Figure 4). At younger ages, cusp tips show higher rates of tissue loss, whereas at older ages, wear becomes more evenly distributed across the entire occlusal surface, with periods of stasis evident (Figures 2 and 3). One individual (640) exhibited a distinctly different wear pattern compared to the others. In this case, the highest degree of tissue loss was observed in occlusal fissures and pits, while distal cusps showed substantially more wear than mesial cusps in both upper and lower first molars. This pattern is atypical compared to the other individuals and suggests potentially pathological tissue loss (Figure 5).

## 4 Discussion

The main outcome of this study is it demonstrates that newly developed techniques in clinical settings using high‐resolution scans of either original teeth or tooth casts are also effective in analyzing tissue loss through time in groups with medium‐high rates of natural physiological (i.e., non‐pathological) wear (O'Toole et al. 2019a, 2020). These findings therefore indicate that such methods are suitable for assessing tooth wear in hunter‐gatherer and mixed‐diet populations, at least across intervals of up to 3 years. Beyond this duration, alignment accuracy may decrease due to more extensive tissue loss. The effectiveness of these methods in medium‐high wear contexts is likely due to the minimal wear on the buccal and lingual surfaces, used to refine 3D mesh alignment, even when there is substantial occlusal tissue loss. In contrast, in clinical settings today, significant occlusal wear is typically accompanied by wear on non‐occluding surfaces, resulting from factors such as erosion (e.g., from acidic fruit drinks or sports drinks) or abrasion (e.g., improper tooth brushing; Oudkerk et al. 2023; Surarit et al. 2023).

In clinical contexts, a physiologically acceptable amount of wear is generally estimated at around 30 μm per year, while individuals with severe tooth wear often exhibit tissue loss several times greater than this baseline (Lambrechts et al. 1989; Rodriguez et al. 2012; Bronkhorst et al. 2023). Even so, most individuals in these clinical studies do not approach the annual average of 250 μm observed in the Yuendumu sample. Studies that have looked at average tissue loss across the whole occlusal surface have also reported significantly less tissue loss in clinical human samples than the present study. For example, Marro et al. (2020) found that molars with macroscopically visible wear progression had an average tissue loss of 2.19 mm^3^ over a two‐year period, while those with no visible differences lost an average of just 0.37 mm^3^. Other studies in a clinical setting typically exhibit similarly low levels of tissue loss, even among those deemed at risk of more severe wear, with annual loss on molars generally below 1 mm^3^ (Pintado et al. 1997; Tantbirojn et al. 2012; O'Toole et al. 2020). The Yuendumu sample showing an average of 4 mm^3^ is therefore substantially more than this, and supports these methods also being used in medium‐high wear samples.

The results also show these methods can quantify common wear patterns observed from research on single time point data, such as functional cusps and locations of known occlusal wear facets (Fiorenza et al. 2011; Kullmer et al. 2012; Oxilia et al. 2018; Towle et al. 2021), generally showing the most wear. There are intervals where other regions of the crown experience similar levels of tissue loss, or minimal loss across the entire occlusal surface is observed. Thus, although these high‐wear areas do exhibit the most tissue loss overall, the progression appears phasic or punctuated, with the tissue loss pattern changing substantially across the occlusal surface during specific stages of dental development. Some other notable trends emerged that warrant further investigation. For example, the two individuals with multiple time‐point comparisons, individuals 549 and 183, showed reduced lower molar wear between the ages of 11 and 14, while upper molars experienced peak wear during the same period. But the overall tissue loss also varies substantially between these two individuals, potentially related to sex‐based differences in tooth wear progression (Richards and Brown 1981; Molnar, McKee, and Molnar 1983; Molnar, McKee, Molnar, and Przybeck 1983; Molnar et al. 1989; Littleton et al. 2013).

The results also suggest that achieving the level of tissue loss typically observed in first molars, where dentin is just exposed (or nearly exposed) on one or more cusps by the time the third molars erupt (e.g., stage 4 of Scott 1979), requires an average annual tissue loss of approximately 0.05 mm^3^/mm^2^ from the time of first molar eruption. Although this rate will vary across individuals and populations, and a larger sample is needed to verify this figure, it offers a useful baseline for future studies to test. Notably, this value is consistent with recent findings from both hunter‐gatherer and archaeological agricultural populations, falling within the lower end of the expected wear range by the time of third molar eruption (e.g., Lovejoy 1985; Mays et al. 2022). However, reported wear levels vary widely across agricultural and hunter‐gatherer populations, and many studies emphasize the importance of factors beyond diet alone when interpreting wear patterns, particularly in comparisons between agricultural, hunter‐gatherer, and transitional populations (Smith 1984; Eshed et al. 2006; Deter 2009; Górka et al. 2015). Larger samples are therefore needed to provide further context and allow more robust comparisons across groups.

The Yuendumu dental casts, previously described as exhibiting varying levels of wear depending on the context and comparative samples (Barrett 1969; Molnar, McKee, and Molnar 1983; Clement et al. 2007; Lee et al. 2021), likely reflect reduced tooth wear compared to earlier generations at Yuendumu, and many purely hunter‐gatherer Aboriginal groups elsewhere in Australia (Brown 1978; Molnar, McKee, and Molnar 1983). However, this reduction is not straightforward, as wear varied substantially among Australian Aboriginal populations with hunter‐gatherer diets, and the Yuendumu sample falls within this variation (Littleton et al. 2013). Coarse/tough or hard foods, as well as grit from food preparation, often dominate wear patterns, meaning a population with a transitional or mixed diet could exhibit similar, or even greater, wear than one subsisting solely on traditional hunter‐gatherer foods. As well as a diet including wild plants and animals, the Yuendumu population at that time also consumed processed foods provided by the government, such as sugar, flour, canned goods and rice (Campbell and Barrett 1953; Middleton and Francis 1976; Molnar, McKee, and Molnar 1983; Corruccini et al. 1990). While the processed foods likely contributed to increased dental pathologies such as caries and crowding (Campbell and Barrett 1953; Cran 1957, 1960), traditional food preparation methods likely introduced grit, and the continued consumption of raw and coarse foods likely drove the observed wear progression (Molnar, McKee, and Molnar 1983; Molnar, McKee, Molnar, and Przybeck 1983; Brown et al. 2011).

But why the pattern of wear changes through dental development requires additional explanations. As Molnar, McKee, Molnar, and Przybeck (1983) observed, occlusal loads shift during arch growth, muscle development, and tooth eruption, leading to complex wear patterns. Permanent first molars take on the major occlusal load during mastication once they erupt, and this is amplified if the deciduous molars are heavily worn (Molnar and Ward 1977). The first molars then continue to do a significant proportion of food mastication until the permanent second molars and premolars come into occlusion several years later. When this does occur, it is possible the first molars could see a reduction in use since the overall occlusal surface areas increase (Brown et al. 2011). However, this is far from clear as, among other influences, occlusal forces and food intake likely increase. More broadly, the change of occlusion during the time of mixed dentition to a fully permanent dentition is complex, with clinically related research showing tooth wear and pathologies are heavily influenced by eruption sequence timings (Kim et al. 2018; Fadel et al. 2022; Stoica et al. 2022). In such contexts, wear is often described as phasic, with periods of progression and stasis (Loomans et al. 2017).

Non‐masticatory activities may also significantly affect tooth wear progression in this sample. Non‐masticatory uses for teeth have been described in Australian Aboriginal people, including piercing, grasping, and shredding type processes (Molnar 1971; Barrett 1977; Clement et al. 2007). This process often includes using teeth as a clamp to hold various materials, but other uses of the dentition are also employed, such as chewing dried animal sinews to soften them before using them as a binding material (Brown et al. 2011). A wide range of other non‐masticatory uses of teeth have been described in the Yuendumu people during this time (Barrett 1977; Brown et al. 2011), which makes it difficult to distinguish masticatory and non‐masticatory wear.

### 4.1 Applications in Biological Anthropology

Technological advances will likely transform the study of dental wear in coming years. High‐resolution 3D models created from serial dental molds or directly from individuals' mouths using intraoral scanners allow precise quantification and visualization of wear progression. Software like *WearCompare* facilitates detailed superimposition of models over time, capturing subtle occlusal changes that go beyond what is feasible to discern from wear scoring methods based on one point in an individual's life (i.e., usually at death in the case of osteological specimens). The most immediate application of these methods in biological anthropology is in assessing tissue loss in contemporary human and non‐human primate populations. Although the number of contemporary human populations (or recent populations that have longitudinal dental casts) with medium‐high rates of physiological tooth wear is likely low, those that do can provide crucial data to calibrate and refine interpretations of single time‐point wear in osteological and paleontological contexts. For example, these methods can refine commonly used wear scoring systems, such as percent of dentine exposure accross the occlusal surface or ordinal grade systems (e.g., Scott 1979; Smith 1984). By assessing how much tissue loss is typically required to move between grades or percentage increase, this will allow for more precise assessment of tooth wear progression and population and dietary differences. They will also help identify which types of wear should be considered atypical or pathological, offering new tools for interpreting and comparing tissue loss across contexts.

Wild non‐human primates also offer promising opportunities for applying these techniques, particularly in field studies that include regular health monitoring or non‐invasive data collection. Scanning or molding dentitions could be easily incorporated into existing protocols, entire dentitions can be captured with intraoral scanners in minutes, or in seconds when only specific areas are targeted. In addition to enabling dietary and behavioral comparisons among populations and species, these data can inform assessments of anthropogenic effects, pathology progression, and environmental influences on tooth wear. They also hold potential as proxies for understanding tissue loss in fossil primate samples. Additionally, insights into how wear progresses in relation to diet (e.g., hard or tough foods) or environmental grit can improve reconstructions of behavior and ecology in fossil hominins, through comparisons with these extant primate samples, patterns that are not easily detectable using standard tooth wear grading methods.

Although the current approach relies on time‐series data (i.e., serial dental casts or 3D meshes), emerging advances in reconstructing original crown morphology may enable broader applications in archaeological and paleontological contexts (e.g., O'Hara et al. 2019; Modesto‐Mata et al. 2024). For example, by digitally reconstructing teeth at various stages of wear, and comparing reconstructed and unreconstructed models, it may become possible to estimate tissue loss progression in archaeological and fossil samples. While most current reconstruction techniques are limited to cusp morphology (O'Hara et al. 2019; Modesto‐Mata et al. 2024, and references therein), growing knowledge of the relationship between enamel‐dentine junction (EDJ) shape and outer enamel form, combined with advances in artificial neural networks and machine learning, suggests that accurate full‐crown reconstructions may soon be feasible. These developments could provide powerful tools for comparing wear progression within and between populations, especially when large samples of individuals across age ranges are available. This, in turn, may offer novel insights into dietary adaptations through applying tissue loss analysis.

The methods employed in this study, originally designed for clinical settings, may be beneficial in paleontological, archeology and primatological studies more broadly by combining other types of wear data, such as occlusal tissue loss data with approaches such as Occlusal Fingerprint Analysis (OFA; Benazzi et al. 2011; Fiorenza et al. 2015; Fiorenza et al. 2024), microwear studies (Krueger et al. 2008; Ungar et al. 2012), and fracture assessments (Towle et al. 2017, 2021; Towle and Loch 2021; Belcastro et al. 2018; Fannin et al. 2023; Towle, Borths, et al. 2024). For instance, general patterns in the present study seem to fit, at least partially, expectations from the literature on Occlusal Fingerprint Analysis (Figure 5), and future research could test this more directly by looking at tissue loss through time focussing on particular occlusal wear facets or areas. Similarly, do microwear features on a single tooth surface vary during periods of minimal versus substantial tissue loss (i.e., during different phases to tooth wear), and are certain cusps especially prone to both pronounced wear and fractures at specific ages? Although integrating different types of tissue loss data is not new (e.g., Teaford and Oyen 1989; Schmidt 2010; Teaford et al. 2017; Towle et al. 2021), deploying new clinical equipment and techniques can refine existing methods and allow more detailed analyses of changes within an individual's lifetime. In much the same way that methodologies from biological anthropology contexts are being adopted in clinical research (e.g., Oxilia et al. 2025; Ungar and Hara 2024), dental anthropologists may find additional tools from clinical dentistry settings.

Finally, recent studies have emphasized the functional role of wear in maintaining occlusion and masticatory efficiency through dynamic reshaping of the occlusal surface (Kaifu et al. 2003; Ungar and M'Kirera 2003; Kaidonis 2008; Benazzi et al. 2013; Lee et al. 2021). Tissue loss progression studies can contribute by providing fine‐grained insights into how dental morphology evolves throughout life. Additionally, the same 3D models used for tissue loss analysis can be incorporated into experimental studies of mastication to explore how and why wear patterns form. As such, these techniques, and the data they generate, may also find broader application in experimental, phylogenetic, and evolutionary studies.

## 5 Conclusions

This study demonstrates that clinical wear‐assessment methods, such as those employed in *WearCompare*, can be effectively adapted for use in biological anthropology contexts to quantify physiological tooth wear. By capturing detailed patterns of occlusal tissue loss in a population with medium‐high natural wear rates, these techniques provide a valuable tool for exploring developmental, dietary, and behavioral influences on tooth wear. Beyond their utility in contemporary populations, these methods offer a promising avenue for refining traditional wear scoring systems in archaeological and paleontological contexts, and integrating with other analytical approaches, enhancing our ability to reconstruct diet and behavior in the fossil record.

## Author Contributions

**Ian Towle:** conceptualization, investigation, writing – original draft, methodology, visualization, writing – review and editing, formal analysis, data curation, project administration, resources. **Luca Fiorenza:** conceptualization, investigation, funding acquisition, visualization, writing – review and editing, formal analysis, project administration, resources, methodology.

## Conflicts of Interest

The authors declare no conflicts of interest.

## Data Availability

The full data set that supports the findings of this study are available in the results section of this manuscript.
